# Medical Matters in 1897

**Published:** 1898-02

**Authors:** 


### EDITORIAL.

The office of THE JOURNAL is 311 and 312 Fitten Building.

Address all communications, and make all remittances payable to THE ATLANTA MEDICAL AND SURGICAL JOURNAL, Atlanta, Ga.

Reprints of articles will be furnished, when desired, at a small cost. Requests for the same should always be made *on the manuscript.*

### MEDICAL MATTERS IN 1897.

The last issue of the Boston *Medical and Surgical Journal* for 1897 presents an instructive and interesting review of the medical history of the past year. To that review we are mainly indebted for what follows:

While 1897 was not marked by great medical events or discoveries, yet progress has been decided and substantial. Further experience has continued to confirm the value of the antitoxin serum of diphtheria, the opponents of which have now little ground to stand upon. The American Pediatric Society made its second annual report (upon 1,700 cases of laryngeal diphtheria) within the year, which closed with the following conclusions as to the effects of this treatment: (1) A great reduction in the mortality of cases treated in the first three days of illness. (2) The lowering of the combined general mortality to a point below that of any former year. (3) The remarkable reduction in mortality of laryngeal cases. (4) The uniform improvement in the results of tracheotomy. (5) The beneficial effects produced on the clinical course of the disease.

In April Professor Koch, of Berlin, announced that he had prepared a new tuberculin which possessed all the virtues of the old without the latter's defects; and he claimed to have established in a large number of cases of lupus and phthisis that its action was much more effective than that of the old product. The general results of experiment with the new tuberculin thus far seem to be of a negative character. More time will be necessary for decided conclusions to be reached.

Considerable activity has been displayed in utilizing the Roentgen rays in medical and surgical diagnosis. In surgery the especial value of the rays has been mainly confined to detecting and correcting deformities, fractures and dislocations and in locating foreign bodies in the tissues or cavities of the body. The rays have been of service also in the diagnosis of internal disease, such as cardiac enlargements, aortic aneurisms, tubercular processes in the lungs, tumors of internal organs, etc.

Alabama and Georgia suffered more during the year from small-pox than any other sections of the country. The epidemic (now, happily, about over) seems to have been started in Pensacola last winter. In May it appeared in Birmingham, and by the last of November 190 cases had occurred, with two deaths. There were about 35 cases in Montgomery and about the same number in Memphis. In and near Atlanta there have been about 240 cases, with three deaths. The disease has been practically confined to the *unvaccinated* negroes. Compulsory vaccination was enforced in all cities where small-pox had appeared and more serious trouble was thus averted. A severe epidemic of small-pox prevailed in Japan in the first six months of 1897; 5,700 cases were reported, with 1,600 deaths. In Cuba small-pox existed all the year. In one week in January there were 990 cases, and 108 deaths in Havana, and at other times there were from 20 to 30 deaths daily in the same city.

Conditions incident to the war in Cuba have favored the continuance and spread of yellow fever, which is present there, more or less, at all times. The proximity of Cuba to our shores and the unsanitary conditions which always prevail there, make the island a grave source of danger to our country. There is little doubt that the late epidemic of yellow fever was imported from Cuba.

"About the 20th of August, at Ocean Springs, Miss., a summer resort on the Gulf coast, the population of which is made up chiefly of excursionists from New Orleans, Mobile and the interior country, it was reported that five or six hundred cases of disease had occurred, which had been pronounced by experts appointed by the boards of health of Mississippi and Louisiana to be dengue. September 6th, Passed Assistant Surgeon Wasdin, of the Marine Hospital Service, performed an autopsy on a case which had died with the symptoms of the prevailing disease and pronounced it yellow fever. An immediate exodus took place of the residents of Ocean Springs to their homes in New Orleans and the surrounding country, and a detention camp was established at a point on the Louisville and Nashville Railroad, twenty miles east of Ocean Springs. John Guiteras, M.D., Professor of General Pathology and Morbid Anatomy, University of Pennsylvania, appointed temporary Acting Assistant Surgeon of the Marine Hospital Service, visited Ocean Springs September 8th, and made the diagnosis of yellow fever in three cases, this diagnosis being subsequently confirmed in one case by an autopsy made by Dr. Wasdin. The large majority of the cases of prevailing fever, Dr. Guiteras pronounced to be dengue."

Within a few days cases of yellow fever appeared in New Orleans, Mobile, Scranton, Biloxi, Edwards, Flomaton, first in the persons of those who had lately fled from Ocean Springs. From these points interior towns in Mississippi, Louisiana, Alabama and Tennessee were infected and new cases continued to break out until the last of November. "From the beginning of the epidemic to December 1st, when it was practically ended, 4,385 cases and 462 deaths had occurred."

In July Professor Sanarelli, of Uruguay, said that he had isolated and cultivated from the blood and tissues of yellow fever patients a bacillus which he considered to be the specific bacillus of this disease. About the same time Surgeon-General Sternberg, of the United States Army, published a further account of a bacillus which he had isolated in 1889, and which he thought probably identical with the bacillus of Sanarelli. Further investigations must decide this question; also whether either is the specific cause of yellow fever.

In the first part of the year famine and the Plague created havoc in India. By the end of March there had been 9,100 cases and 7,600 deaths in Bombay alone. It had about died out by September.

